# The ability to manipulate ROS metabolism in pepper may affect aphid virulence

**DOI:** 10.1038/s41438-019-0231-6

**Published:** 2020-01-01

**Authors:** Mengjing Sun, Roeland E. Voorrips, Martijn van Kaauwen, Richard G. F. Visser, Ben Vosman

**Affiliations:** Plant Breeding, Wageningen University & Research, P.O. Box 386, 6700 AJ Wageningen, Netherlands

**Keywords:** Biotic, Virulence, Plant breeding, Biodiversity

## Abstract

*Myzus persicae* has severe economic impact on pepper (*Capsicum*) cultivation. Previously, we identified two populations of *M. persicae*, NL and SW, that were avirulent and virulent, respectively on *C. baccatum* accession PB2013071. The transcriptomics approach used in the current study, which is the first study to explore the pepper−aphid interaction at the whole genome gene expression level, revealed genes whose expression is differentially regulated in pepper accession PB2013071 upon infestation with these *M. persicae* populations. The NL population induced ROS production genes, while the SW population induced ROS scavenging genes and repressed ROS production genes. We also found that the SW population can induce the removal of ROS which accumulated in response to preinfestion with the NL population, and that preinfestation with the SW population significantly improved the performance of the NL population. This paper supports the hypothesis that *M. persicae* can overcome the resistance in accession PB2013071 probably because of its ability to manipulate plant defense response especially the ROS metabolism and such ability may benefit avirulent conspecific aphids.

## Introduction

Aphids, which belong to the order of Hemiptera, are one of the most destructive plant pests worldwide. Among the more than 4000 described aphid species, over 100 species are reported as economically important pests and are able to damage plant health^1^. Aphid infestation can result in direct damage such as chlorosis, necrosis, wilting, defoliation and, more importantly, indirect damage resulting from aphid transmitted viruses^2^. Most aphid species reproduce asexually under suitable conditions, which leads to rapid population expansion and therefore to difficulties in population control^1^.

Given the fact that aphids have severe negative effects on crop cultivation, the frequent use of chemical pesticides is the major management strategy^3^. However, with growing concern about the negative impact of pesticides on the environment, integrated pest management such as promoting aphid resistant varieties is more and more encouraged^4^. Several resistant crop varieties have been applied to alleviate aphid problems, such as melon varieties resistant to cotton aphid *Aphis gossypii*^5^, lettuce varieties resistant to the black current-lettuce aphid *Nasonovia ribisnigri*^6^ and soybean varieties resistant to the soybean aphid *Aphis glycines*^7^. Although using resistant varieties is a beneficial strategy to control aphids, the durability of crop resistance is threatened by the evolution of new aphid biotypes which have overcome the resistance^8^. Understanding the interaction between aphids and their host plants, including how the resistance response is induced in resistant plants and how aphids adapt to host plant resistance, may help to keep crop resistance more durable during agricultural application.

Plants may induce defense responses against aphid feeding. The defense responses induced in resistant plants include calcium influxes^9^, accumulation of reactive oxygen species (ROS)^10^, phloem occlusion by specific proteins^11,12^ and callose deposition^13^. ROS have been suggested to play an important role in plant defense responses against biotic stresses. They not only may have a direct toxic effect on aphids^14^, but have also been suggested to mediate defense gene activation and interact with other signaling components^15^. Accumulation of ROS in plants could enhance aphid resistance^16^ while impairment of ROS production reduces aphid resistance, e.g. makes the plant more susceptible^17^. For example, greenbug (*Schizaphis graminum*) feeding on resistant sorghum induced the expression of peroxidase which caused ROS production^18^. Moreover, induction of ROS activity was very high in the resistant wheat after the infestation of Russian wheat aphid (*Diuraphis noxia*)^19^, while it was low in wheat after the infestation of another *D. noxia* population that had overcome the resistance^20^. As ROS are involved in a large network associated with plant defense responses, it is conceivable that their levels may be affected/regulated by multiple enzymes. It has been proposed that ROS can be produced by various enzymes, such as NADPH oxidases^21^, peroxidases^22^ and oxalate oxidase^23^, and may be removed by ROS-scavenging enzymes like catalase^24^ and superoxide dismutase^25^, or by antioxidants like glutathione^26^.

Pepper (*Capsicum* spp.) is one of the most important and widely cultivated horticultural crops. However, pepper cultivation is hampered by aphids and the viruses they transmit^27^. One accession of *Capsicum baccatum* (PB2013071) has been recently identified as a good resistance source to a population of *Myzus persicae* (the NL population^13^). The resistance of this accession was later found to be (partly) overcome by another population of *M. persicae* (the SW population)^10^. The *M. persicae* populations performed the same on the plants of susceptible accession PB2013046, while the SW population performed significantly better than the NL population on the plants of accession PB2013071 ^10^. The susceptibility of accession PB2013046 to both populations was reflected in electrical penetration graph (EPG) recordings that showed similar feeding activities for both aphid populations on that accession. Additionally, neither aphid population induced ROS on PB2013046. The better performance of the SW population on plants of accession PB2013071 was reflected in longer phloem feeding and a much weaker induction of the defense response. To better understand why the two *M. persicae* populations, NL (incompatible interaction) and SW (compatible interaction), performed so differently on *C. baccatum* accession PB2013071, we (1) analyzed gene expression in the compatible and incompatible interaction to identify differentially expressed genes (DEGs) that specifically responded to the virulent and avirulent aphid population respectively, with an emphasis on genes involved in ROS production and scavenging; (2) studied ROS accumulation to investigate whether the SW population of *M. persicae* is able to promote plant susceptibility by suppressing ROS accumulation; and (3) studied the ability of both *M. persicae* populations to induce plant susceptibility for conspecific aphids.

## Results

### Transcriptome profiling

Pepper accession PB2013071 shows resistance to aphids of the *M. persicae* NL population, but is susceptible to aphids of the *M. persicae* SW population. On this accession aphids of the NL population show a reduced survival and a poor reproduction. Aphids of the SW population encounter much fewer problems in survival and reproduction^10^.

RNA isolated from plants of accession PB2013071 treated with aphids of NL population (NL-infested), SW population (SW-infested) or empty clip cages (control) for 6 h was subjected to RNA-seq analysis. On average, 7.7 GB clean data (6.7−9.2 GB) per sample were generated (detailed information in Table S1). All the datasets have been submitted to European Nucleotide Archive (ENA) (accession number: PRJEB35311).

**Fig. 1:**
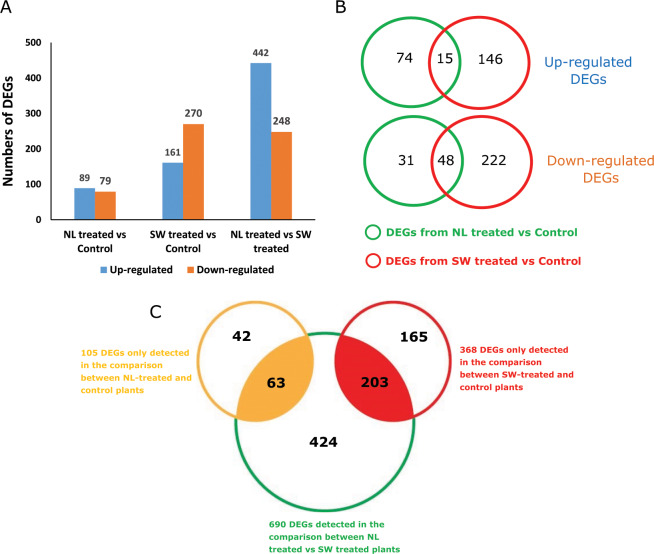
Differentially expressed genes (DEGs) identified in response to two *M. persicae* populations. Plants received clip cage with aphids of the NL (NL-treated) or SW (SW-treated) population, control plants received an empty clip cage. After a 6-h incubation, leaf disks were harvested and analyzed using RNAseq. The criteria used for assigning significance were: FDR ≤ 0.01 and |log2FoldChange| ≥ 1. **a** Number of upregulated or downregulated DEGs; **b** number of DEGs specifically or coexpressed in comparison between control and NL-treated plant as well as in comparison between control and SW-treated plant. **c** Venn diagram of DEGs that specifically respond to the feeding by the NL or the SW population. DEGs were first compared between the NL-treated vs. control plants and SW-treated vs. control plants. One hundred and five genes were differently regulated in the comparison between NL-treated and control plants (yellow circle) and 368 genes were differently regulated in the comparison between SW-treated and control plants (red circle). The lists of 105 and 368 genes were then compared with the list of 690 DEGs that were detected in the direct comparison between NL-treated and SW-treated plants. The 63 and 203 genes that appeared in both comparisons were considered here to specifically respond to the feeding by the NL and the SW populations respectively.

Using a false discovery rate (FDR) of 0.01 and a |log_2(_FoldChange)| ≥ 1, there were 168, 431 and 690 DEGs detected between NL-infested and control plants, between SW-infested and control plants and between NL-infested and SW-infested plants, respectively (Fig. 1a, Table S2).

To validate the results obtained from RNA-seq, the expression level of six genes was measured by QPCR in all nine samples. For each gene, the fragments per kilobase of transcript per million fragments (FPKM) values of transcriptome data exhibited similar expression patterns for all the three treatments compared with the QPCR results (Fig. S1). The correlation coefficient between RNA-seq and QPCR ranged from 0.86 to 1, indicating that the RNA-seq expression data are reliable.

### Genes differentially expressed in response to feeding by both aphid populations

Among all the DEGs identified from the comparison between NL-infested and control plants and between SW-infested and control plants, only 15 genes were upregulated and 48 genes were downregulated in both comparisons (Fig. 1b, Table S3). GO enrichment analyses of the 15 common upregulated genes responding to both aphid populations showed that five and six genes were predicted to be involved in cellular components (GO:0005575) and biosynthetic processes (GO:0009058), respectively. Among the 48 downregulated genes, genes involved in photosynthesis (GO:0009522, GO:00095223, GO:0009535, GO:0016168) were over-represented (26 genes out of 48), of which genes encoding chlorophyll a-b binding proteins were the main group (21 genes).

### Differentially expressed genes specific for feeding of the NL or the SW population

Of all genes that were differentially regulated in response to feeding of the two aphid populations, most genes are regulated in a population-specific way, showing that transcriptional responses are largely aphid population dependent (Fig. 1b).

When compared with control plants, 105 genes were only significantly up- or down-regulated in the plants that were treated with aphids of the NL population, and 368 genes only after feeding by the SW population (Fig. 1b, Table S4). In a direct comparison between plants that were treated with the NL and SW population, 690 genes were differentially expressed. This list was used to narrow down the number of genes specifically involved in response to different aphid populations. There were 63 genes responding specifically to feeding by the NL population and 203 specifically responding to feeding by the SW population (Fig. 1c, Table S5). GO enrichment analyses of the 63 genes responding to the NL population indicated that they were mainly involved in oxidoreductase activity (GO:0016491, 19 genes), ion binding (GO:0043167, 19 genes), biosynthetic process (GO:0009058, 11 genes) and response to stress (GO:0006950, 10 genes). The 203 genes specifically responding to SW population were predicted to be mainly involved in different activities such as working in integral component of membrane (GO:0016021, 36 genes), oxidation−reduction processes (GO:0055114, 24 genes), ATP binding (GO:0005524, 19 genes), protein kinase activity (GO:0004672, 18 genes) and phosphorylation (GO:0006468, 18 genes).

### Differentially expressed genes involved in oxidoreductase activity

We previously observed that aphids of the NL population induce a strong accumulation of ROS, while the SW population induces only a very weak ROS response in plants of PB2013071^10^. As we knew from the previous study that differences in ROS accumulation were apparent 6 h after infestation, the transcriptome analysis of the current study was carried out after 6 h of infestation. We looked for differences in plant DEGs involved in ROS metabolism, especially in DEGs that specifically responded either to the NL or to SW population (Table S5). Given that ROS production and scavenging is a dynamic oxidation−reduction process, we looked for the DEGs that are assigned with predicted functions related with oxidation−reduction process, which is reflected in the GO annotations GO:0016491, GO:0016709, GO:0016717, GO:0055114 and GO:0003824.

Among the 63 DEGs that specifically respond to infestation by the NL population, there were 18 upregulated and 4 downregulated genes with oxidoreductase activity (Table 1). Similarly, among the 203 DEGs responding only to the SW population, 14 up- and 16 downregulated were annotated as having oxidoreductase activity (Table 2). Among all DEGs involved in oxidoreductase activity (Tables 1 and 2), only one gene was present in both lists. Peroxidase 12 was upregulated in NL-infested plants while downregulated in SW-infested plants.

**Table 1:** Differentially expressed genes (DEGs) involved in oxidation−reduction processes in resistant *C. baccatum* PB2013071 specifically responding to the NL aphid population.

Gene ID	Control—average FPKM	NL-treated—average FPKM	log2FoldChange	Up−down-regulation	FDR	Gene annotation
rna5965	113	475	2.07	Up	1.88E-26	Peroxidase N1
rna10318	9013	23,158	1.36	Up	1.86E-08	Peroxidase 12
rna10320	1003	2596	1.37	Up	7.59E-07	Partial Peroxidase 12
rna8690	1	25	4.07	Up	0.0048	Peroxidase 5
rna15242	1570	4540	1.53	Up	0.00034	Linoleate 9S-lipoxygenase 5
rna19402	99	328	1.73	Up	0.00036	Carotenoid 9,10(9',10')-cleavage dioxygenase 1
rna29042	66	219	1.74	Up	0.0062	DMR6-Like oxygenase
rna34704	78	279	1.84	Up	1.30E-08	Omega-6 fatty acid desaturase
rna29538	136	465	1.78	Up	0.00074	Omega-6 fatty acid desaturase
rna21402	52	164	1.66	Up	0.0014	Delta(12)-fatty-acid desaturase
rna28453	64	180	1.49	Up	0.0018	l-ascorbate oxidase
rna24520	1176	2963	1.33	Up	0.00084	Cytochrome P450 76A1
rna10853	667	1733	1.38	Up	0.0033	Cytochrome P450 82A3
rna28819	604	1588	1.39	Up	0.0014	3-oxoacyl-[acyl-carrier-protein] reductase
rna19820	157	463	1.56	Up	0.0059	NAD(P)H: quinone oxidoreductase
rna29543	1241	3277	1.4	Up	0.0025	7-alpha-hydroxysteroid dehydrogenase
rna21511	69	215	1.64	Up	0.0061	Arogenate dehydrogenase
rna25969	68	155	1.19	Up	0.0069	Aldehyde dehydrogenase
rna11857	3276	794	−2.04	Down	2.03E-13	Geranylgeranyl diphosphate reductase
rna16367	810	1121	−1.22	Down	1.54E-06	NADP-dependent glyceraldehyde-3-phosphate dehydrogenase
rna22917	68	18	−1.90	Down	0.000033	Cytochrome P450 83B1
rna14689	279	97	−1.52	Down	0.00042	Chlorophyllide a oxygenase

The criteria used for assigning significance were: FDR ≤ 0.01 and |log_2_FoldChange| > 1

**Table 2:** Differentially expressed genes (DEGs) involved in oxidation−reduction processes in resistant *C. baccatum* PB2013071 specifically responding to the SW aphid population.

Gene ID	Control—average FPKM	SW-treated—average FPKM	log2FoldChange	Up−down-regulation	FDR	Gene annotation
rna4866	1154	5384	2.22	Up	1.14E-44	Catalase
rna26330	2357	6131	1.38	Up	6.61E-30	Alanine aminotransferase
rna8250	161	538	1.74	Up	4.10E-22	Cytochrome P450 71A6
rna12135	37	141	1.91	Up	2.31E-09	Cytochrome P450 CYP82D47
rna19682	37	173	2.24	Up	5.39E-09	Ferric reduction oxidase 5
rna5789	106	342	1.68	Up	1.23E-08	Cinnamoyl-CoA reductase
rna7913	286	969	1.76	Up	1.24E-07	4-coumarate-CoA ligase 2
rna20326	165	506	1.62	Up	3.00E-07	Ferric reduction oxidase 6
rna2114	101	250	1.31	Up	3.21E-07	Galactinol−sucrose galactosyltransferase 4
rna21997	23	143	2.63	Up	1.94E-06	Flavonol synthase/flavanone 3-hydroxylase
rna8249	38	124	1.70	Up	0.000046	Cytochrome P450 71A6
rna14917	246	526	1.10	Up	0.00021	Quinone-oxidoreductase homolog
rna31209	193	392	1.02	Up	0.0030	Geraniol 8-hydroxylase
rna10118	331	704	1.09	Up	0.0061	Cytochrome P450 71A1
rna25419	416	88	−2.23	Down	1.61E-24	Cytochrome P450 86A1
rna5501	5302	1710	−1.63	Down	7.44E-23	Polyphenol oxidase B
rna17709	897	302	−1.57	Down	2.43E-22	Endoplasmic reticulum oxidoreduction-1
rna27753	224	40	−2.50	Down	2.72E-20	DMR6-Like oxygenase
rna10848	863	235	−1.87	Down	7.46E-19	Allene oxide synthase
rna14282	10875	2580	−2.08	Down	4.37E-18	Beta-carotene hydroxylase 2
rna32427	379	126	−1.60	Down	2.07E-17	Alkane hydroxylase MAH1
rna35370	1659	797	−1.06	Down	5.16E-16	Alcohol-forming fatty acyl-CoA reductase
rna19821	1894	762	−1.32	Down	1.02E-11	NAD(P)H:quinone oxidoreductase
rna23236	10323	1032	−3.32	Down	2.59E-09	Protochlorophyllide reductase
rna28288	214	85	−1.35	Down	0.000029	Cytochrome P450 CYP736A12
rna5811	59467	28426	−1.07	Down	0.000055	Oxygen-evolving enhancer protein 1
rna23410	304	147	−1.05	Down	0.000056	Delta(8)-fatty-acid desaturase
rna20968	355	147	−1.27	Down	0.000057	9-divinyl ether synthase
rna10318	9013	4152	−1.12	Down	0.000112	Peroxidase 12
rna18808	29	4	−2.70	Down	0.000140	Cytokinin dehydrogenase 3

The criteria used for assigning significance were: FDR ≤ 0.01 and |log2FoldChange| >1

### Effect of aphid population SW on reactive oxygen species (ROS) metabolism

In order to explore the effect of the SW population on plant ROS metabolism, we analyzed ROS in pepper leaves of accession PB2013071 after five different treatments, including preinfestations with the NL population.

**Fig. 2:**
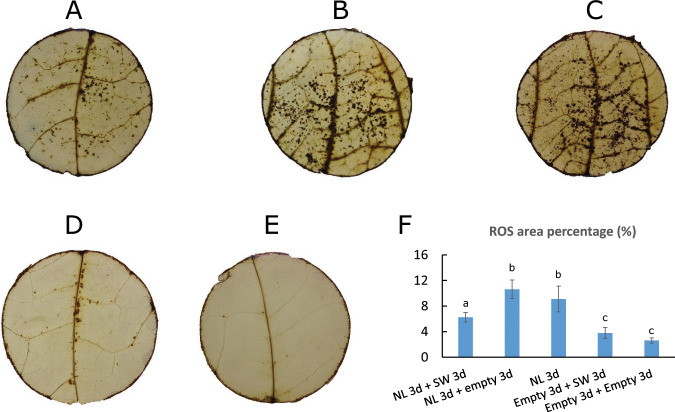
ROS accumulation in leaves of pepper accession PB2013071 in response to *M. persicae* populations NL and SW. DAB staining was used to show ROS accumulation after different treatments. **a** Leaf disk after a 3-day infestation with the NL aphids population. **b** Leaf disk after a 3-day infestation with NL aphids followed by 3 days with an empty cage. **c** Leaf disk after a 3-day infestation with NL aphids followed by 3 days with the SW aphid population. **d** Leaf disk after 3 days with an empty cage followed by 3 days SW population. **e** Leaf disk after 6 days with an empty cage. **f** shows the percentage of stained DAB area calculated per leaf disk under the clip cage area. Bars represent means ± SD. Different letters indicate statistically significant differences between treatments (LSD-test, *P* < 0.05).

Plants of accession PB2013071 showed a strong ROS accumulation after 3 days of feeding by aphids of the NL population (Fig. 2a), and this accumulation could not be effectively eliminated by the plants themselves after a further 3-day period with empty cages (Fig. 2b). However, this induced ROS accumulation was mostly eliminated after a subsequent infestation of SW population (Fig. 2c). The reduction in ROS accumulation was reflected by a significantly lower percentage of DAB staining area on the leaf (Fig. 2f, LSD-test, *P* < 0.05).

Although the presence of the SW aphid population induced a weak ROS accumulation in plants of PB2013071 (Fig. 2d), there was no significant difference in the percentage of stained area between SW-infested leaf and control leaf that received an empty clip cage (Fig. 2d–f, LSD-test, *P* > 0.05).

### Effect of preinfestation with aphid populations on a subsequent infestation

Besides the RNA-seq analysis and ROS accumulation assays, we also carried out bio-assays to explore whether defense responses induced by the NL population can affect SW performance and whether the manipulation of plant defenses by the SW population could benefit NL population.

**Fig. 3:**
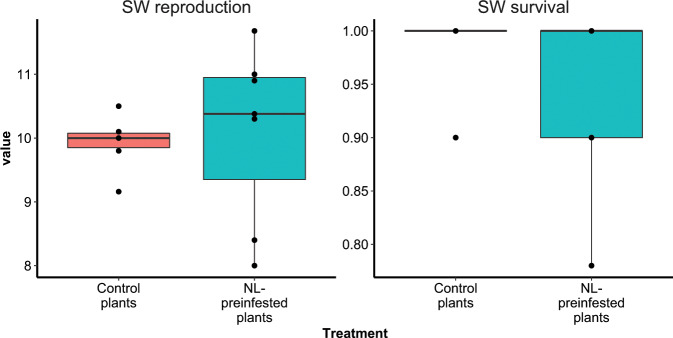
Performance of the SW population on plants of pepper accession PB2013071. Plants were preinfested with *M. persicae* of the NL population (NL-preinfested plants) or received an empty clip cage (control plants) for 3 days, after which the aphids were removed. Next, the plants received clip cages with *M. persicae* of the SW population. Two phenotyping parameters were used: average number of next-generation nymphs produced per SW living adult after 12 days (left panel), and the fraction of SW aphids initially put on a plant that survived 12 days (right panel). Seven biological replicates (plants) were used per treatment and are presented as black dots in the box plots. For both phenotyping parameters, no significant difference between the treatments was found (*t* test, *P* > 0.05).

**Fig. 4:**
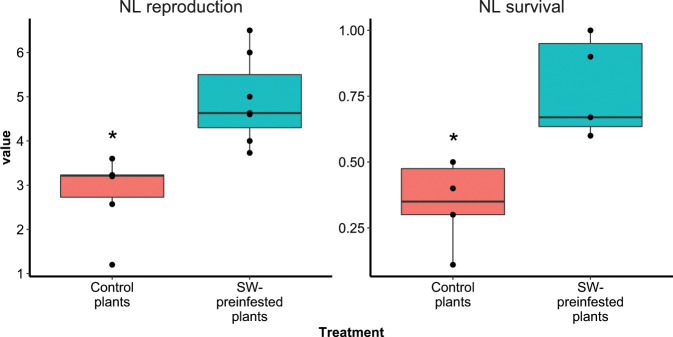
Performance of the NL *M. persicae* population on plants of pepper accession PB2013071. Plants were preinfested with the SW population (SW-preinfested plants) or received an empty clip cage (control plants) for 3 days, after which the aphids were removed. Next, the plants received clip cages with aphids of the NL population. Two phenotyping parameters were used: average number of next-generation nymphs produced per living NL adult after 12 days (left panel), and the fraction of NL aphids initially put on a plant that survived 12 days (right panel). Seven biological replicates were used per treatment and are presented as black dots in box plots. For both phenotyping parameters, a significant difference between the treatments was found (*t* test, *P* < 0.01).

Figure 3 shows the effect of preinfestation with the NL population on the performance of the SW population. There was no significant difference in aphid survival and reproduction between living on NL preinfested plants and living on control plants (*t* test, *P* > 0.05). When the situation was turned around and plants were first infested with aphids of the SW population and the effect on the performance of aphids of the NL population was measured, the outcome was different. Aphids of *M. persicae* population NL showed a significantly higher survival and produced significantly more next-generation nymphs on SW preinfested plants than on control plants of PB2013071 (Fig. 4, *t* test, *P* < 0.01). Preinfestation with the SW population made it possible for the NL aphids to increase survival from 0.35 ± 0.14 to 0.78 ± 0.17 and to improve reproduction from 2.84 ± 0.87 to 4.92 ± 1.01 nymphs per original aphid.

## Discussion

### Plants show a very different transcriptional response to different conspecific aphid populations

In our previous study we identified two *M. persicae* populations, NL and SW, which are avirulent and virulent on pepper accession PB2013071, respectively. The NL population had difficulties with phloem ingestion and induced a strong defense response, including callose deposition and ROS accumulation in plants of PB2013071, and as a result it was not able to colonize this accession. In contrast, the SW population was able to start sustained phloem intake very easily and only induced a very mild defense response in this accession^10^. In our transcriptome analysis, plants of accession PB2013071 show a very different response to the two *M. persicae* populations. Of all genes that are differentially regulated in response to feeding of the two aphid populations, 88% are regulated in a population-specific way (Fig. 1b), and many more genes are regulated only by the SW population than by the NL population (Fig. 1c; 203 vs. 63). This might be simply an effect of the longer sustained feeding of the SW population^10^, or it could be due to their greater ability to manipulate plant defenses at the gene expression level.

Gene expression among different plant genotypes (resistant/susceptible) in response to one aphid population/clone has been studied frequently^28–30^. Also there are several studies showing the response of one plant genotype to different aphid species^31–33^. However, we found only one report of specific plant gene expression induced by different populations/biotypes of the same aphid species. That report describes the response of wheat to two different biotypes of the Russian wheat aphid^34^. It shows that most wheat genes are regulated in a biotype-specific way, and that one biotype can regulate many more genes than the other, which is similar to our transcriptome results. To our best knowledge, the current study is the first one to explore the interaction between pepper and conspecific aphid populations at the whole genome gene expression level.

To identify genes regulated differently between infestations with the two aphid populations, we used a combination of two approaches. One was to find a set of DEGs comparing noninfested with NL-population-infested plants, and a second set of DEGs comparing noninfested plants with SW-population-infested plants, and then filter for genes occurring in only one of these sets (Fig. 1b). The other approach was to find DEGs in the direct comparison between plants infested with the NL population and plants infested with the SW population. By looking for the DEGs occurring in the datasets of both methods, we found 63 and 203 genes specifically regulated by infestation with the NL population and SW population, respectively (Fig. 1c).

### Differentially expressed genes are involved in defense signaling pathways in pepper

When plants are able to induce defense responses against aphids, multiple signaling pathways may be elicited, including phytohormone-induced pathways^35^. Some genes that are involved in defense signaling pathways are found to specifically respond to feeding by the NL and SW populations, respectively (Table S5). For example, linoleate 9S-lipoxygenase (9-LOX, rna15242) is specifically induced by the NL population, and the gene expression level in NL-infested plants is three times higher compared with the one in control plants as well as in SW-infested plants. The 9-LOX gene in pepper can induce responsive genes of salicylic acid (SA) and jasmonic acid pathways (JA), accumulate ROS and therefore enhance resistance to several microbial pathogens^36^. Several genes that are involved in abscisic acid (ABA) pathway are only regulated by infestation with the SW population. The ABA-insensitive 5 (rna20904) gene is upregulated while ABA receptors (rna12809 and rna23680) are downregulated after SW infestation. It has been reported that overexpression of ABA receptors can promote resistance to bacteria^37^, while a loss of function of the ABA-insensitive 5 gene can impair ROS-scavenging activities in *Arabidopsis*^38^. The upregulation of ABA-insensitive 5 and downregulation of ABA receptors in pepper after feeding by the SW population may help to promote plant susceptibility. It has been shown that a virulent bacterial effector promotes plant susceptibility in *Arabidopsis* through manipulating the ABA pathway^39^. As genes involved in the ABA pathway are only regulated by the SW population, it is possible that the SW population promotes the colonization on PB2013071 by targeting ABA pathway-related genes.

### Differentially expressed genes are involved in ROS accumulation and scavenging in pepper

Previously we found a correlation between ROS accumulation and plant resistance. The plants of accession PB2013046 could not induce ROS accumulation after the infestation by either the NL or SW population, and they were susceptible to both *M. persicae* populations. The plants of accession PB2013071 induced a strong and mild ROS accumulation respectively after the infestation by the NL and SW population, and they were more resistant to the NL than the SW population^10^. Based on this work, we speculated that some of the *M. persicae* population-specific DEGs in plants of accession PB2013071 may be related with ROS induction and scavenging. We identified DEGs involved in ROS metabolism by looking for genes predicted to be involved in oxidation−reduction processes. Among the DEGs that specifically responded to infestation with the NL population are four upregulated genes encoding peroxidases (Table 1), which may be involved in ROS production^40^. Peroxidase-dependent ROS production has been described in several studies before^22,41^. In *C. annuum*, one peroxidase (CaPO2) has been reported to be required for ROS generation and this gene has been found to enhance plant resistance against bacteria^42^ and fungi^43^. So far there have been no reports on the involvement of peroxidase in insect resistance in pepper. The peroxidase 5 (rna8690), which is only upregulated by NL aphids, shares high similarity with CaPO2 in amino acid sequence (Fig. S2), suggesting that CaPO2 and rna8690 might be orthologous genes in *C. annuum* and *C. baccatum*. In addition, the activity of peroxidase 12 has been found to be responsible for ROS accumulation in maize and thereby to enhance resistance to the fungus *Ustilago maydis*, and inhibition of peroxidase 12 increased the infection rate of *U. maydis*^44^. In our RNA-seq results, peroxidase 12 (rna10318) is the only peroxidase gene that is downregulated in the plants treated with SW aphids, which has been validated by QPCR (Fig. S1). However, maize and pepper are very distantly related species and peroxidase 12 of maize shares only 50% sequence similarity with that of pepper at the protein level (Fig. S3); therefore, the function of peroxidase 12 may have changed and further work is needed to establish its exact role in pepper. NADPH oxidases have also been suggested to cause ROS accumulation in plant-biotic interactions^21,45^, and higher levels of NADPH oxidase activity have been seen in resistant than in susceptible wheat and maize infested with aphids^46^. A mutation of the NADPH oxidase *RBOHD* gene in *Arabidopsis* results in decreased ROS accumulation and causes increased *M. persicae* susceptibility^47^. The *RBOHD* gene in PB2013071 is up- and down-regulated upon infestation by the NL and SW population, respectively, but does not pass the criteria of |log_2_(FoldChange)| in our analysis (0.33 and 0.71, respectively), which suggests peroxidase-mediated ROS production may play a more important role in the pepper−aphid interaction than NADPH oxidase-mediated ROS production, at least after 6 h of infestation.

Catalase (rna4866) is the most significantly upregulated gene with oxidation−reduction process in plants of PB2013071 that specifically responded to the infestation with the SW aphid population (Table 2). The expression difference of this gene in aphid-infested pepper has also been validated by QPCR (Fig. S1). Catalases are among the fastest enzymes that convert H_2_O_2_ to oxygen and water as they do not require a reductant^24^. Suppression of catalase has been found to enhance ROS levels in response to biotic stress in various plant species such as tobacco^48^ and sorgum^49^. Conversely, higher levels of catalase activity have been shown to increase susceptibility to pathogens^50,51^ and also to *M. persicae*^52^. There are three main isoforms of catalases: class I, II and III^24,53^. Class I catalases are highly expressed in mature leaves and include *Cat1* of *Nicotiana plumbaginifolia*^53^ and *N. tabacum*^54^, which showed about 96% sequence identity to the catalase transcript (rna4866) of PB2013071 (Fig. S4). Based on the role of catalase in ROS metabolism and plant defense, the five times higher expression level of catalase (rna4866) in leaves of PB2013071 infested by SW aphids might be one of the most important reasons why the SW population is able to colonize on pepper accession PB2013071. Serine-glyoxylate aminotransferase and alanine aminotransferase are also found to be upregulated in PB2013071 treated by SW aphid population. They are relevant for glutathione biosynthesis and therefore are involved in ascorbate and glutathione cycle that is the major nonenzymatic ROS scavenging process^55^. The increased activity of serine-glyoxylate aminotransferase has been found to be related with a decrease of ROS accumulation^56^ and also has been found in the plants that interact with pathogens in a compatible way^57^. Additionally, the expression levels of two ferric reduction oxidase genes are also increased. Ferric reduction oxidases participate in the process of H_2_O_2_ production and scavenging^58^, and one ferric reduction oxidase has been shown to block ROS accumulation in *Arabidopsis*^59^. Therefore, besides catalase the higher expression of aminotransferase and ferric reduction oxidase genes may also contribute to the suppression of ROS accumulation in PB2013071 after the infestation of SW *M. persicae* population.

In summary, a strong ROS accumulation is induced in PB2013071 after feeding by the NL population, which is most likely caused by the upregulation of several genes promoting ROS accumulation, including peroxidases and NADPH oxidases. Several ROS-scavenging genes are upregulated in PB2013071 after feeding by the SW population, including catalase and aminotransferases, which may explain the mild ROS accumulation in this acccession.

### The ability to suppress ROS accumulation may explain why the pepper resistance is overcome by a virulent *M. persicae* population

In previous experiments a strong defense response involving ROS accumulation was induced by aphids of the NL population on plants of PB2013071, but only a very weak response by the SW population^10^. In the current study we observed in the SW-infested plants of PB2013071 the downregulation of several genes for enzymes known to have a role in ROS production, and the upregulation of some genes for enzymes known to have a role in ROS scavenging. The ability of the SW population to manipulate ROS metabolism of pepper plants by the RNA-seq data is also reflected by the ROS accumulation experiment combined with preinfestation with the avirulent NL aphid population (Fig. 2). The results show that SW aphids can induce removal of most of the ROS accumulated in response to the preinfestion with NL aphids. The balance between ROS production and scavenging may determine the strength of the plant defense response^60^. In several studies ROS accumulation has been observed in plants upon interaction with pathogens or insects, and differences in this ROS accumulation are linked with differences in plant resistance^14,19^. Additionally, ROS accumulation in host plants is in several cases linked with the virulence or avirulence of pathogens, such as fungi^61^, bacteria^62^ and nematodes^60^. Only very few studies have been published linking differences in ROS accumulation to differences in virulence among pest insects^63,64^. Our results on ROS accumulation after preinfestation with an avirulent aphid population clearly show the ability of virulent aphids to suppress ROS formation and break down existing ROS. Further validation of this could come from experiments in which key genes involved in ROS accumulation are silenced by virus-induced gene silencing (VIGS) or knocked out by CRISPR. The VIGS technique has been used in the functional analysis of some pepper genes, however until now only in *C. annuum*^65–67^. So far no VIGS has been reported in *C. baccatum*. A disadvantage of VIGS in insect studies is also the patchy nature of the silencing, making it easy for aphids to find nonaffected spots on leaves. Alternatives like producing CRISPR knockouts have also not been developed yet, due to poor transformability of *Capsicum* species^68^.

### The ability of virulent aphids to manipulate plant defenses may benefit avirulent aphids

Preinfestation with the NL population does not significantly change the plant response to the subsequent infestation with the SW population (Fig. 3). Based on the fact that plants of PB2013071 induce a strong defense response after infestation with the NL population (Fig. 2)^10^, the noneffect of NL preinfestation to the performance of SW population suggests that the SW population can cope with the plant defense responses induced by the other conspecific aphid.

Preinfestation with the SW population resulted in a significantly better performance of the NL population (Fig. 4), which is reflected in a higher survival of original aphids and higher number of next-generation nymphs produced. The phenomenon that feeding by a virulent aphid population induces susceptibility to a conspecific avirulent population has been also observed in the interaction between soybean and soybean aphid *Aphis. glycines*^69^ as well as in the interaction between lettuce and the black current-lettuce aphid *Nasonovia ribisnigri*, although the mechanism of this induced susceptibility remained unclear^70^. Based on our study we can hypothesize that the benefit caused by virulent aphid populations to the conspecific avirulent aphid population might be due to manipulation of plant defense responses, especially the ROS metabolism. The expression level of pepper genes such as catalase that are induced by virulent aphids may remain high after the preinfestation, which might help the following avirulent aphids to start phloem feeding successfully. As we removed the virulent aphids, probably when they were still feeding, it is possible that secreted virulent effectors continued to induce specific pepper genes.

## Materials and methods

### Plant material and aphid populations

*C. baccatum* accessions PB2013071 and PB2013046 were obtained from the collection of Plant Breeding, Wageningen University & Research, NL and are described in ref. ^13^.

Two weeks after sowing, seedlings were transplanted into 14 cm pots with potting compost and grown in a standard greenhouse at 19−21 °C, 60–70% relative humidity with an L16:D8 photoperiod at Unifarm, Wageningen University & Research, NL. Plants were watered every other day. Seven-week-old plants of PB2013071, which were still in vegetative stage, were used in all the experiments.

The two *M. persicae* populations (NL and SW) used were described previously^10^. The NL population is avirulent on pepper accession PB2013071, while the SW population is virulent. Both populations were reared on susceptible *C. baccatum* accession PB2013046 in cages in different greenhouse compartments under the same conditions as used for growing the plants.

### RNA-seq experiment

Plants of accession PB2013071 were infested for 6 h with aphids of the NL (NL-infested) or SW population (SW-infested), or received an empty clip cage (control), after which RNA was extracted (Fig. S5). Each treatment included three biological replicates and three plants were pooled for each replicate. All the used plants were grown in the same greenhouse compartment at the same time. The first three fully expanded leaves from the top of every plant each received one clip cage either with ten randomly selected apterous adults of the NL or SW population, or one empty clip cage. After 6 h treatment, aphids were gently brushed away from the leaves, and leaf disks under clip cages from one biological replicate (three plants) were quickly sampled and pooled. Leaf disks were flash-frozen in liquid nitrogen and stored at −80 °C until use. Total RNA was extracted using the RNeasy Plus Mini Kit (Qiagen, the Netherlands) according to the suppliers’ recommendations. RNA quality and quantity were evaluated by NanoDrop 1000 V.3.7 (Thermo, USA), Qubit fluorometric quantitation (Thermo, USA) and agarose gel electrophoresis before sending for RNA-seq analysis.

Library construction and sequencing were performed by Novogene Bioinformatics Technology Co., Ltd (Beijing, China). After cDNA library construction, sequencing was performed on an Illumina HiSeq 2500 system (Illumina, USA) and 2×150 bp pair-ended reads were generated. In total, at least 6 GB data were generated per replicate.

### Bioinformatic analysis of RNA-seq data

After the quality of raw data was evaluated by FASTQC^71^, sequence reads of each biological replicate were mapped to the PBC81 *C. baccatum* reference genome (http://peppergenome.snu.ac.kr/)^72^ using STAR^73^. The number of reads per gene was counted with Salmon (https://combine-lab.github.io/salmon/)^74^ and transcript abundance was calculated using the FPKM (fragments per kilobase of transcript per million fragments) method^75^. The SARTools pipeline^76^, which is based on DESeq2 package in R^77^, was employed to detect DEGs between the control treatment and the NL-infested treatment or SW-infested treatment, as well as for the direct comparison between NL- and SW-infested plants. In this pipeline, an FDR analysis^78^ was implemented to correct the *P* values of the multiple *t* tests in these comparisons. Genes with a FDR ≤ 0.01 and |log_2_(FoldChange)| ≥ 1 were classified as differentially expressed. Blast2GO v5 Basic (https://www.blast2go.com/)^79^ was used to carry out gene ontology (GO) analysis to predict the function of DEGs.

### Gene expression validation

Six genes with differential expression levels based on the RNA-seq data were selected for validation with quantitative real-time PCR. The pepper *UBI3* gene (ubiquitin-conjugating protein) was used as an internal reference for normalization of gene expression^80^. Gene-specific primers were designed using Primer3Plus (www.bioinformatics.nl/cgi-bin/primer3plus/primer3plus.cgi) and are listed in Table S6. Each first-strand cDNA was synthesized using the iScriptTM cDNA Synthesis Kit (Bio-Rad, USA) with 1 µg RNA. Quantitative RT-PCR was performed in duplicate as described previously^13^. QPCR products were sequenced to verify that the correct fragment was amplified in PB2013071. The relative transcription level of each gene was calculated using the 2^−ΔΔCt^ method^81^.

### Reactive oxygen species (ROS) accumulation experiment

In order to explore whether the SW population is able to suppress ROS accumulation induced in PB2013071, five different treatments were designed. Three leaves each with one clip cage from five independent biological replicates (plants) were used for each treatment. Four of the five treatments consisted of a preinoculation with either the NL population (15 randomly selected apterous adults in a clip cage) or an empty cage for 3 days. In both cases the clip cage, and if present the aphids, were then removed and replaced at the same place with either an empty clip cage or a clip cage containing 15 apterous adults of the SW population. After this second infestation, lasting again 3 days, the leaves were harvested for observation. In this way four combination treatments were applied (preinfestation–infestation): NL–empty, NL–SW, empty–SW and empty–empty. The fifth treatment consisted of only the preinfestation with NL after which the leaves were harvested directly.

After the treatment leaf disk areas under the clip cage were collected and aphids were gently brushed away if needed. DAB (3,3′-Diaminobenzidine) staining of leaf disks was performed as described^10^. The photos of mounted glass slides with leaf disks were taken with a Canon EOS 100D camera (Canon Inc., Japan). The percentage of the area of brown polymerized deposits, which reflect ROS accumulation on each leaf disk, was quantified using ImageJ^82^. The average percentage from three clip cages that were collected from the same plant was used as the data for one biological replicate. Data were transformed as arcsin(sqrt(*x*)) to stabilize the residual variance. The significance of the difference in level of ROS accumulation between the five treatments was evaluated using ANOVA with the LSD test (*P* < 0.05).

### Effect of preinfestation on subsequent aphid performance

A no-choice assay with clip cages was carried out to study whether preinfestation by the NL population had an effect on the performance of SW population on pepper plants of accession PB2013071 and vice versa.

Seven plants of accession PB2013071 were preinfested with three clip cages containing 15 randomly selected apterous adults of the NL population. Another seven plants were similarly preinfested with the SW population. Two control groups of seven plants received three empty clip cages. The cages were placed on first three fully expanded leaves from the top of every plant. Cages were kept on the plants for 3 days, after which the original adult aphids together with offspring were gently removed with a soft brush. Every preinfested plant then received three clip cages containing five 1-day-old nymphs of the aphid population that was different from the population used for preinfestation: NL preinfested plants received SW aphids and vice versa. Plants of two control groups were infested with aphids of either the NL or SW population. The clip cages were put on the same spots on the leaves where the removed clip cages had been. After 12 days the living and dead aphids as well as the next-generation nymphs produced in each clip cage were counted. The observations from three clip cages per plant were combined. Aphid survival and reproduction were determined as described in ref. ^10^. For statistical analysis, aphid survival and reproduction were transformed as arcsin(sqrt(*x*)) and sqrt(*x*), respectively, to stabilize the residual variance. A *t* test was used to compare the difference in aphid survival and reproduction on preinfested and control plants (*P* < 0.05).

## Supplementary information

Table S1

Table S2

Table S3

Table S4

Table S5

Table S6

Figure S1

Figure S2

Figure S3

Figure S4

Figure S5
