# Supplementary material for: The ability to manipulate ROS metabolism in pepper may affect aphid virulence
Source: Hortic Res. 2020 Jan 1;7:6. doi: 10.1038/s41438-019-0231-6 (PMC6938493; doi:10.1038/s41438-019-0231-6)
Supplement: Supplementary file 1 — Table S1 [file 41438_2019_231_MOESM1_ESM.docx]

| **Table S1. Sequencing and assembly statistics for the 9 transcriptome samples of PB2013071 after different treatments.** | | | | | | | | | |
| --- | --- | --- | --- | --- | --- | --- | --- | --- | --- |
| Sample ID | No. of raw reads (×10^6^) | No. of clean reads (×10^6^) | Raw base (G) | Clean base (G) | Effective rate (%) | GC content  (%) | Q30 (%) | Percentage of uniquely mapped  reads(%) | Percentage of multiply mapped  reads(%) |
| Control-1 | 25.59 | 24.55 | 7.7 | 7.4 | 95.93 | 42.48 | 94.31 | 87.18 | 5.46 |
| Control-2 | 23.03 | 22.49 | 6.9 | 6.7 | 97.68 | 42.31 | 93.09 | 88.18 | 6.10 |
| Control-3 | 24.54 | 23.86 | 7.4 | 7.2 | 97.22 | 42.89 | 94.09 | 87.25 | 6.02 |
| NL-infested-1 | 26.01 | 25.57 | 7.8 | 7.7 | 98.31 | 41.77 | 92.73 | 88.81 | 6.03 |
| NL-infested-2 | 26.19 | 25.68 | 7.9 | 7.7 | 98.06 | 42.41 | 92.96 | 88.94 | 5.55 |
| NL-infested-3 | 27.36 | 26.73 | 8.2 | 8.0 | 97.73 | 41.85 | 93.09 | 88.45 | 5.87 |
| SW-infested-1 | 27.37 | 27.07 | 8.2 | 8.1 | 98.91 | 42.83 | 91.91 | 89.27 | 5.81 |
| SW-infested-2 | 31.64 | 30.73 | 9.5 | 9.2 | 97.13 | 42.55 | 93.76 | 88.14 | 5.51 |
| SW-infested-3 | 25.35 | 24.49 | 7.6 | 7.3 | 96.63 | 42.01 | 93.14 | 87.77 | 6.19 |
| Note: ‘Control’ plants received empty clip cages. ‘NL-infested’ and ‘SW-infested’ plants received clip cages with 10 randomly selected adults of the NL and SW *M. persicae* population, respectively. All the treatments lasted for 6 hour, and each sample consisted of leaves from 3 individual plants. | | | | | | | | | |
