# Supplementary material for: The ability to manipulate ROS metabolism in pepper may affect aphid virulence
Source: Hortic Res. 2020 Jan 1;7:6. doi: 10.1038/s41438-019-0231-6 (PMC6938493; doi:10.1038/s41438-019-0231-6)
Supplement: Supplementary file 6 — Table S6 [file 41438_2019_231_MOESM6_ESM.docx]

**Table S6. Primer sequences used in real-time PCR.**

| **Gene ID/Name** | **Forward sequence (5’-3’)** | **Reverse sequence (5’-3’)** |
| --- | --- | --- |
| rna30017 | GCCGTGAAGATGTGGGTCAATGA | TGAGTTACGCCAGACTACCTGAGTA |
| rna4866 | ATCCACTCGATGTGACAAAAACT | GAGTAATAAACCCCTGGAACCAC |
| rna19925 | TACAACCCGCAGAACATCAA | TGTGTTCCCGTTCCTGTGTT |
| rna22768 | GTTGTTGAAGTGCCTAAACTTGG | GGAGCTTAGCGAGTTGGTAG |
| rna10318 | GTTACTTGATGGATCAGCAGGAG | AAGTTGAGTCCGTCCTTTCTTC |
| rna3577 | AGCATTATGGGTGCCTATACCTT | GACAAAGGTCATCTTCTTGATGG |
| *UBI3* | TGTCCATCTGCTCTCTGTTG | CACCCCAAGCACAATAAGAC |
