# Supplementary figures and images for: The ability to manipulate ROS metabolism in pepper may affect aphid virulence
Source: Hortic Res. 2020 Jan 1;7:6. doi: 10.1038/s41438-019-0231-6 (PMC6938493; doi:10.1038/s41438-019-0231-6)

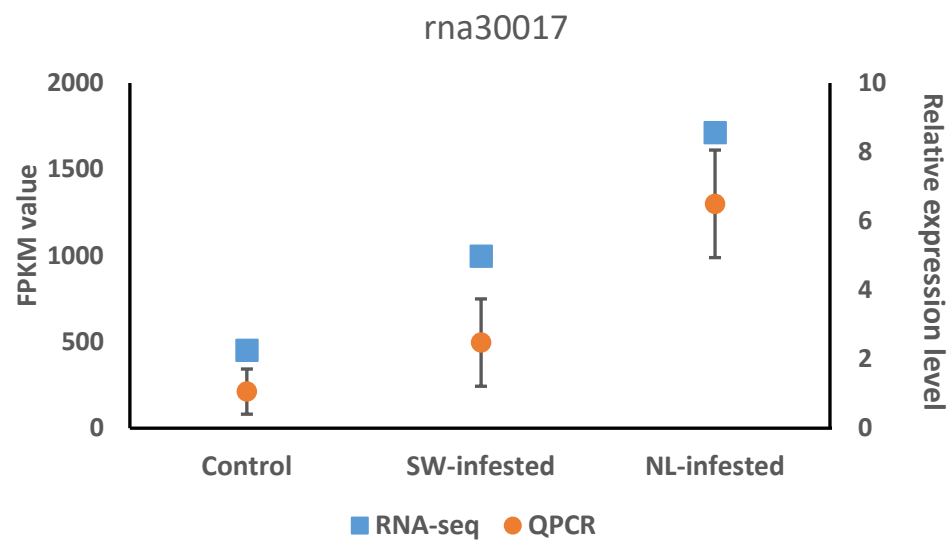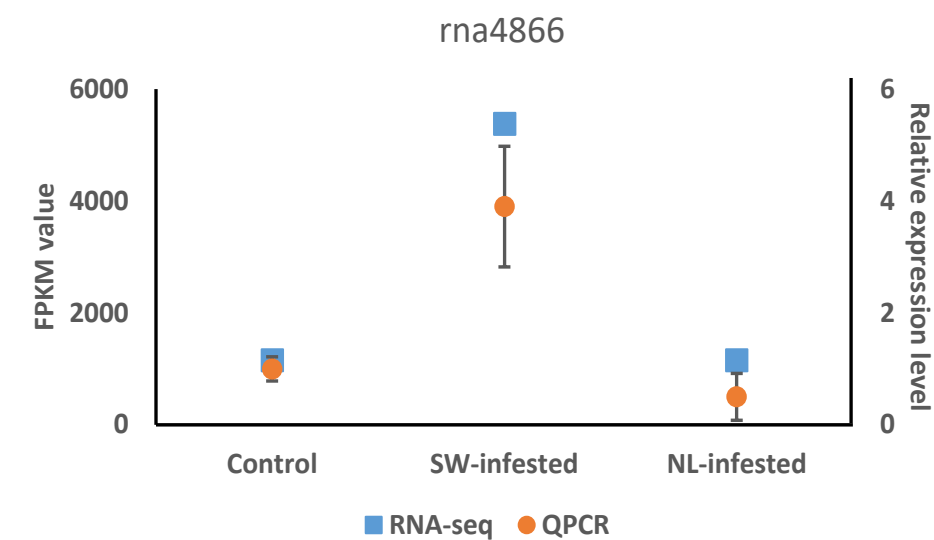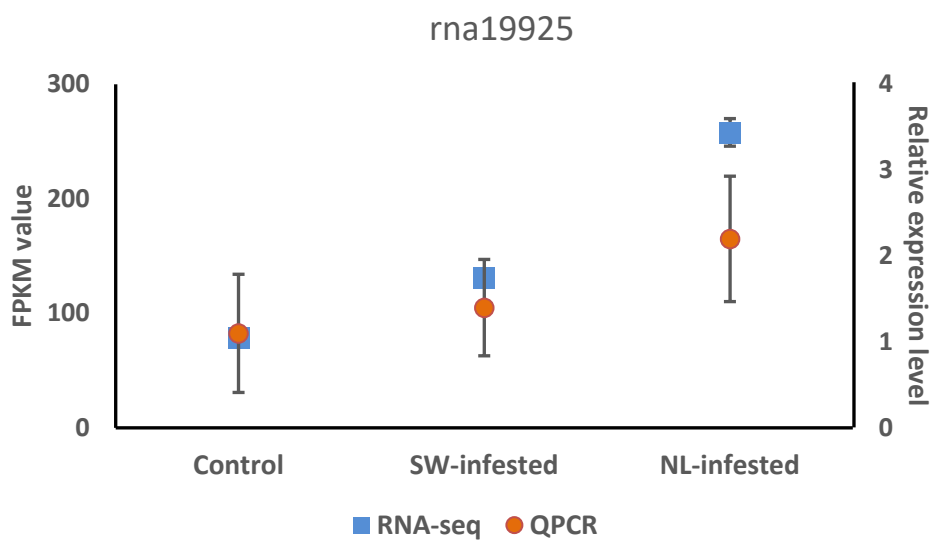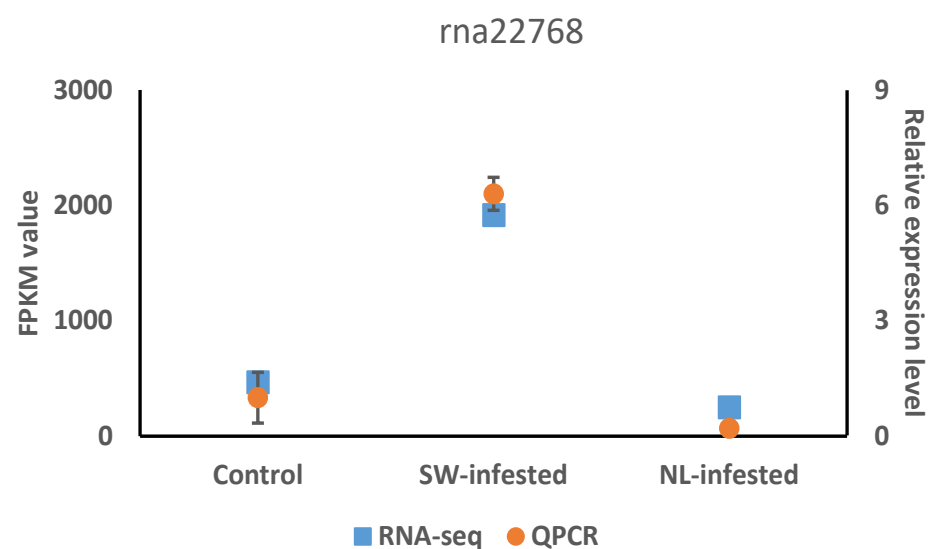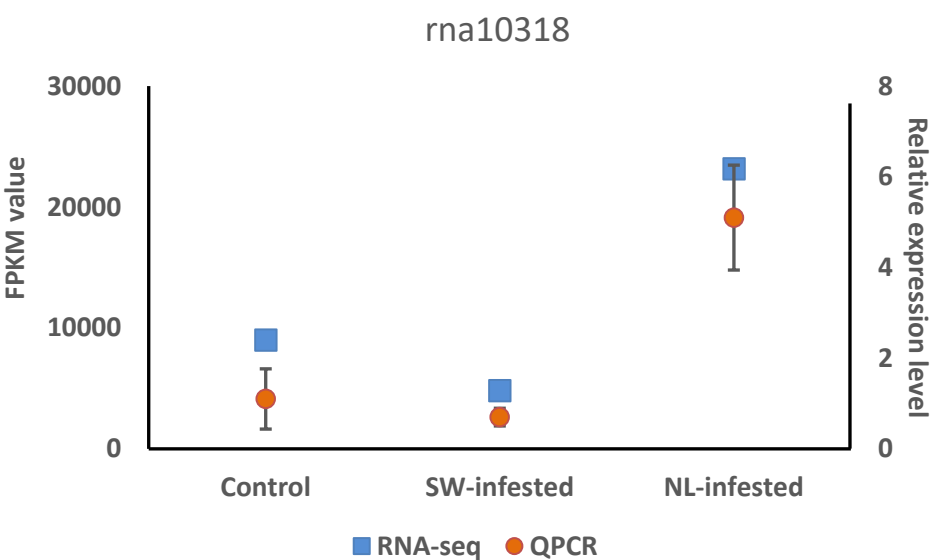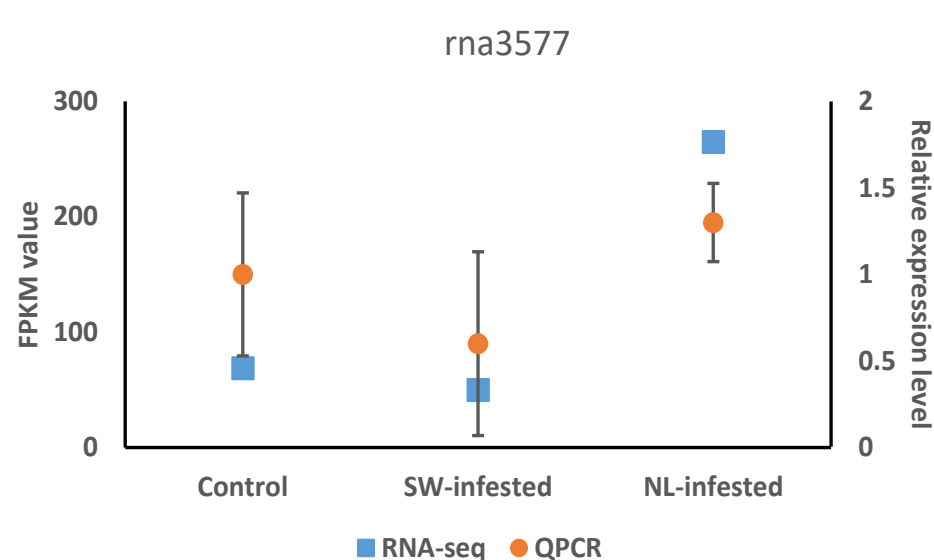

Supplement: Supplementary file 7 — Figure S1 [file 41438_2019_231_MOESM7_ESM.pdf]
