# Supplementary material for: The ability to manipulate ROS metabolism in pepper may affect aphid virulence
Source: Hortic Res. 2020 Jan 1;7:6. doi: 10.1038/s41438-019-0231-6 (PMC6938493; doi:10.1038/s41438-019-0231-6)
Supplement: Supplementary file 8 — Figure S2 [file 41438_2019_231_MOESM8_ESM.pdf]

```
                       1        10        20        30        40        50        60
CaPO2                  MSISNNSFAAVAAIFSLVLLCSMQCHAQLSSTFYDRACPNALNTIRKSVRQAVSAERRMA
Peroxidase5_rna8690_   ..........................................................MA

                                70        80        90       100       110       120
CaPO2                  ASLIRLHFHDCFVQGCDASILLDETPTIVSEKTALPNLGSVRGYGIIEDAKRELEKTCPG
Peroxidase5_rna8690_   ASLIRLHFHDCFVQGCDASILLDETPTIVSEKTALPNLGSVRGYGIIEDAKRELEKTCPG

                               130       140       150       160       170       180
CaPO2                  IVSCADILAVAARDASTLVGGPSWTVKLGRRDSTTASHTLAETDLPGPFDPLTRLISGFA
Peroxidase5_rna8690_   VVSCADILAVAARDASTLVGGPSWTVKLGRRDSTTASHTLAETDLPGPFDPLTRLISGFA

                               190       200       210       220       230       240
CaPO2                  KKGLSTRDMVALSGSHSIGQAQCFLFRDRIYSNGTDIDAGFASTRRRRCPQEDQNGNLAP
Peroxidase5_rna8690_   NKGLSTRDMVALSGAHSIGQAQCFLFRDRIYSNESDIDAGFASTRRRQCPQEDQNGNLAP

                               250       260       270       280       290       300
CaPO2                  LDLVTPNQLDNNYFKNLRQRKGLLQSDQVLLSGGSTDDIVLEYSNSPRAFASDFAAAMIR
Peroxidase5_rna8690_   LDLVTPNQLDNNYFKNLRQRKGLLQSDQVLLSGGSTDDIVLEYSNSPRAFASDFAAAMIR

                               310       320
CaPO2                  MGDISPLTGSNGIIRTVCGAIN
Peroxidase5_rna8690_   MGDISPLTGSNGIIRMVCGAIN
```
