# Supplementary material for: The ability to manipulate ROS metabolism in pepper may affect aphid virulence
Source: Hortic Res. 2020 Jan 1;7:6. doi: 10.1038/s41438-019-0231-6 (PMC6938493; doi:10.1038/s41438-019-0231-6)
Supplement: Supplementary file 9 — Figure S3 [file 41438_2019_231_MOESM9_ESM.pdf]

```
                          1        10        20        30        40        50        60        70        80
Peroxidase12_maize_       MAAATSSRRPAAALLVLAAAVVALAAEGARYPPLAPGLSFDFYKKSCPKAESIVREFLASAVRQNVGLAAALIRLHFHDC
Peroxidase12_rna10318_    ..............MRVKHSLILFKIYEAQRPALVKGLSWSFYQSSCPQLESIIKKRLEKQIKDDVGQAAGLLRLHFHDC

                                   90       100       110       120       130       140       150       160
Peroxidase12_maize_       FVQGCDASILLDATPTQPSEQQSPPNLTLRPAAFKAVNDIRARLDQACGRVVSCADIVALAARESVALGGGPAYKLPLGR
Peroxidase12_rna10318_    FVQGCDSSVLLDGSAGGPSEQTAIPNLTLRKKSFKIIDDLRKRIQAECGQVVSCSDITAIAARDSVVLTGGPKYDVPLGR

                                  170       180       190       200       210       220       230       240
Peroxidase12_maize_       RDGLAPASNAAVLAALPPPTSKVPTLLSFLAKINLDVTDLVALSGGHTVGIAHCGSFDNRLFPTQDPTLNKFFAGQLYRT
Peroxidase12_rna10318_    KDGLNFATEQATIDNLVAPFANTTTILDRLAAKGLDATDAVALSGAHTIGISHCTSFTERLYPNQDRTMDKTFANNLKRS

                                  250       260       270       280       290       300       310       320
Peroxidase12_maize_       CPTNATVNTTANDVRTPNAFDNKYYVDLLNREGLFTSDQDLLTNATTRPIVTRFAVDQDAFFHQFVYSYVKMGQVNVLTG
Peroxidase12_rna10318_    CPTADSNNTVNMDIRSPNVFDNKYYVDLMNRQGLFTSDQDLYTDKRTRGIVTSFAVNQSLFFEKFVIGMIKMGQLNVLTG

                                  330       340       350       360
Peroxidase12_maize_       SQGQVRANCSARNGAAAGDSDLPWSSVVIETVADAAGSLVL
Peroxidase12_rna10318_    GQGEIRNRCDRRN....KDKKVDIATVVEELEETFSALF..
```
