# Supplementary material for: The ability to manipulate ROS metabolism in pepper may affect aphid virulence
Source: Hortic Res. 2020 Jan 1;7:6. doi: 10.1038/s41438-019-0231-6 (PMC6938493; doi:10.1038/s41438-019-0231-6)
Supplement: Supplementary file 10 — Figure S4 [file 41438_2019_231_MOESM10_ESM.pdf]

```
                1        10        20        30        40        50        60        70
NpCAT1   .......PSSAFNSPFCTTNSGAPVFNNNSSLTVGARGPVLLEDYHLVEKLANFDRERVPERVVHARGASAKGFFEVTHD
NtCAT1   MDPYKYRPSSAFNSTFCTTNSGAPVFNNNSSLTVGARGPVLLEDYHLVEKLANFDRERVPERVVHARGASAKGFFEVTHD
rna4866  MDPYKYRPSSAFNSPFCTTNSGAPVWNNNSSLTVGARGPVLLEDYHLVEKLANFDRERIAERVVHARGASAKGFFEVTHD

           80        90       100       110       120       130       140       150
NpCAT1   ITHLTCADFLRAPGVQTPVIVRFSTVIHERGSPETLRDPRGFAVKFYTREGNFDLVGNNFPVFFIRDGMKFPDMVHALKP
NtCAT1   ITHLTCADFLRAPGVQTPVIVRFSTVIHERGSPETLRDPRGFAVKFYTREGNFDLVGNNFPVFFIRDGMKFPDMVHALKP
rna4866  ITHLTCADFLRAPGVQTPVIVRFSTVIHERGSPETLRDPRGFAVKFYTREGNFDLVGNNFPVFFIRDGMKFPDMVHALKP

          160       170       180       190       200       210       220       230
NpCAT1   NPKSHIQENWRVLDFFSHVPESLHMFTFLFDDIGIPQDYRHMDGSGVHTFTLINKAGKSTYVKFHWKPTCGVKSLLEDEA
NtCAT1   NPKSHIQENWRVLDFFSHVPESLHMFTFLFDDIGIPQDYRHMDGSGVHTFTLINKAGKSTYVKFHWKPTCGVKSLLEDEA
rna4866  NPKSHIQENWRVLDFFSHHPESLHMFTFLFDDIGIPQDYRHMDGSGVHTFTLINKAGKSTYVKFHWRPTCGVKSLLEEEA

          240       250       260       270       280       290       300       310
NpCAT1   ARVGGANHSHATQDLYDSIAAGNYPEWKLFIQTMDPDHEDRFDFDPLDVTKTWPEDILPLQPVGRLVLNKNIDNFSNENE
NtCAT1   ARVGGANHSHATQDLYDSIAAGNYPEWKLFIQTMDPDHEDRFDFDPLDVTKTWPEDILPLQPVGRLVLNKNIDNFFNENE
rna4866  IRVGGANHSHATQDLYDSIAAGNYPEWKLFIQTMDPAHEDRFDFDPLDVTKTWPEDIFPLQPVGRLVLNKNIDNFFNENE

          320       330       340       350       360       370       380       390
NpCAT1   QLAFCPSIVVPGVYYSDDKMLQTRIFSYSDTQRYRLGPNYLQLPANAPKCAHHNNHYDGSMNFMHRDEEIDYFPSRYDPV
NtCAT1   QLAFCPSIVVPGVYYSDDKMLQTRIFSYSDTQRYRLGPNYLQLPANAPKCAHHNNHYDGSMNFMHRDEEIDYFPSRYDPV
rna4866  QLAFCPSIVVPGVYYSDDKMLQTRIFSYSDTQRYRLGPNYLQLPANAPKCAHHNNHYDGSMNFMHRDEEIDYFPSRYDPV

          400       410       420       430       440       450       460       470
NpCAT1   RHAEKYPIPSTMCTGKREKCVIQKENNFKQPGERYRSFTPDRQERFIRRWVETLSDPRITYEIRSIWISYWSQADKSLGQ
NtCAT1   RHAEKYPIPSTMCTGKREKCVIQKENNFNQPGERYRSFTPDRQERFIRRWVEALSDPRITYEIRSIWISYWSQADKSLGQ
rna4866  RHAENYPIPTTVCSGKREKCIIQKENNFQQPGERYRSFSADRQERFIRRWVEALSDPRITYEIRSIWISYWSQADKSLGQ

          480
NpCAT1   KLASRLNVRPSI
NtCAT1   KLASRLNVRPSI
rna4866  KLASRLNI....
```
