# Supplementary material for: The ability to manipulate ROS metabolism in pepper may affect aphid virulence
Source: Hortic Res. 2020 Jan 1;7:6. doi: 10.1038/s41438-019-0231-6 (PMC6938493; doi:10.1038/s41438-019-0231-6)
Supplement: Supplementary file 11 — Figure S5 [file 41438_2019_231_MOESM11_ESM.pdf]

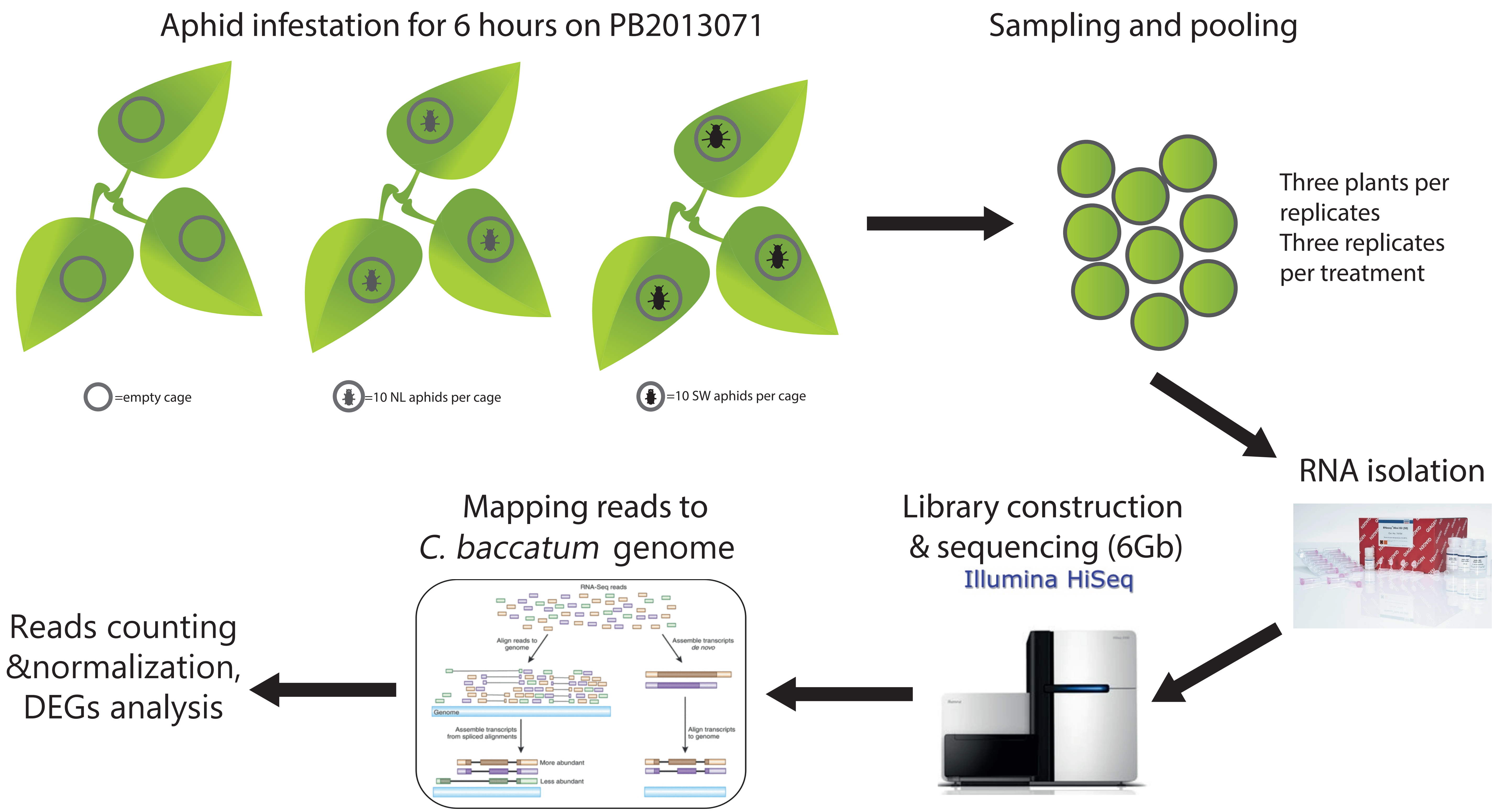

Aphid infestation for 6 hours on PB2013071
Sampling and pooling
=empty cage
=10 NL aphids per cage
=10 SW aphids per cage
Three plants per replicates
Three replicates per treatment
RNA isolation
Library construction & sequencing (6Gb)
Illumina HiSeq
Mapping reads to *C. baccatum* genome
RNA-Seq reads
Align reads to genome
Assemble transcripts *de novo*
Genome
Assemble transcripts from spliced alignments
Align transcripts to genome
More abundant
Less abundant
Reads counting &normalization, DEGs analysis
